# Retrospective Study of Fishery Interactions in Stranded Cetaceans, Canary Islands

**DOI:** 10.3389/fvets.2020.567258

**Published:** 2020-10-21

**Authors:** Raquel Puig-Lozano, Antonio Fernández, Eva Sierra, Pedro Saavedra, Cristian M. Suárez-Santana, Jesús De la Fuente, Josué Díaz-Delgado, Ana Godinho, Natalia García-Álvarez, Daniele Zucca, Aina Xuriach, Marina Arregui, Idaira Felipe-Jiménez, Francesco Consoli, Pablo J. Díaz-Santana, Simone Segura-Göthlin, Nakita Câmara, Miguel A. Rivero, Simona Sacchini, Yara Bernaldo de Quirós, Manuel Arbelo

**Affiliations:** ^1^Veterinary Histology and Pathology, Atlantic Center for Cetacean Research, University Institute of Animal Health and Food Safety (IUSA), Veterinary School, University of Las Palmas de Gran Canaria, Las Palmas of Gran Canaria, Spain; ^2^Department of Mathematics, University of Las Palmas de Gran Canaria, Las Palmas of Gran Canaria, Spain; ^3^TVMDL Texas A&M, Veterinary Medical Diagnostic Laboratory, College Station, TX, United States; ^4^Life and Health Sciences Research Institute (ICVS), School of Medicine, University of Minho, Braga, Portugal; ^5^ICVS/3B's - PT Government Associate Laboratory, Braga/Guimarães, Portugal

**Keywords:** fisherman aggressions, peracute underwater entrapment, entanglement, bycatch, Bryde's whale, Atlantic spotted dolphin, longline hooks, minke whale

## Abstract

Estimating cetacean interactions with fishery activities is challenging. Bycatch and chronic entanglements are responsible for thousands of cetacean deaths per year globally. This study represents the first systematic approach to the postmortem investigation of fishery interactions in stranded cetaceans in the Canary Islands. We retrospectively studied 586 cases necropsied between January 2000 and December 2018. Of the cases with a known cause of death, 7.4% (32/453) were due to fishery interactions, and the Atlantic spotted dolphin (*Stenella frontalis*) was the most affected species [46.9% (15/32)]. Three types of fishery interactions were recognized by gross findings: bycatch [65.6% (21/32)], chronic entanglements [18.8% (6/32)], and fishermen aggression [15.6% (5/32)]. Among the bycaught cases, we differentiated the dolphins that died because of ingestion of longline hooks [23.8% (5/21)] from those that died because of fishing net entrapments [76.2% (16/21)], including dolphins that presumably died at depth due to peracute underwater entrapment (PUE) [37.5% (6/16)], dolphins that were hauled out alive and suffered additional trauma during handling [43.8% (7/16)], and those that were released alive but became stranded and died because of fishery interactions [18.7% (3/16)]. Gross and histologic findings of animals in each group were presented and compared. The histological approach confirmed gross lesions and excluded other possible causes of death. Cetaceans in good-fair body condition and shallow diving species were significantly more affected by fishery interactions, in agreement with the literature. Low rates of fishery interactions have been described, compared with other regions. However, within the last few years, sightings of entangled live whales, especially the minke whale (*Balaenoptera acutorostrata*) and Bryde's whale (*B. edeni*), have increased. This study contributes to further improvement of the evaluation of different types of fishery interactions and may facilitate the enforcement of future conservation policies to preserve cetacean populations in the Canary Islands.

## Introduction

Fishery activities are a major threat to cetacean populations globally (1). Bycatch is a major cause of mortality and poses the highest widespread risk (2, 3). Detection of bycatch among cetaceans is challenging, as there are typically no pathognomonic lesions (4, 5).

Bycaught dolphins in gillnets or trawls are often reportedly healthy individuals in good body condition, with pathological findings that are usually consistent with peracute underwater entrapment (PUE) (5). The most common findings in this type of bycatch are net cuts and impressions on the skin (mainly over the head, but also affecting the flippers and body), changes in the lung (edema, multifocal emphysema, and atelectasis), recently ingested food, reddish or bulging eyes, congestion, and disseminated gas bubbles (6). Other bycatch findings include those produced by fishermen, such as gunshots, stabs over the body, or amputations to disentangle the animal from fishing nets, and abdominal cuts to sink the carcasses (5, 7). To identify bycatch as a cause of death, it is essential to rule out other possible causes of death.

Another type of fishery interaction is chronic entanglement with an active net, or with abandoned, lost or otherwise discarded fishing gear, which form part of the marine debris, and cause ongoing “ghost-fishing” for years (8, 9). At least 14 cetacean species have been reportedly entangled, and 97% of the cases were attached to fishing gear (10). Entanglements are considered a global threat, which international entanglement response and monitoring programs urge (11).

The Canary Islands are located in the region of Macaronesia, close to the north-western coast of Africa. With up to 30 cetacean species (Biocan—Banco del Inventario Natural de Canarias^1^, seven mysticetes and 23 odontocetes, the Canary Islands hold the greatest cetacean biodiversity among European territories, and its fisheries are mainly artisanal. The Atlantic Center for Cetacean Research has been monitoring the health of free-ranging cetaceans stranded in the Canary Islands over the last 20 years. The three most stranded species are the Atlantic spotted dolphin (*Stenella frontalis*), the short-beaked common dolphin (*Delphinus delphis*), and the striped dolphin (*Stenella coeruleoalba*) (12, 13).

The aim of this study was to retrospectively investigate the prevalence and most common pathological findings of each type of fishery interaction, among stranded cetaceans in the Canary Islands. Our results will likely aid the promotion of adequate conservation policies in the archipelago and improve detection of this anthropic threat.

## Materials and Methods

Postmortem examinations of 586 cetaceans stranded along the coasts of the Canary Islands were performed from January 2000 to December 2018, following standardized protocols (14). No experiments were performed on live animals. Permission for the handling of stranded cetaceans was granted by the Spanish Ministry of Environment.

For each necropsied cetacean, the epidemiology of the stranding (i.e., location and date); life history data (i.e., species, growth development, sex, and gonad maturation); body condition; and decomposition code, were systematically recorded. Growth development categories (neonate, calf, juvenile, subadult, and adult) were extrapolated from the osteological characteristics of stranded cetaceans in the Canary Islands (15). Gonad maturation was determined, based on histological gonadal examination (16). Body condition (very poor, poor, fair, or good) was estimated, based on anatomical landmarks (17). The decomposition code (1-very fresh, 2-fresh, 3-moderate autolysis, 4-advanced autolysis, and 5-very advanced autolysis) was determined following the classification of IJsseldijk et al. (18). During the necropsy, lesions were described and photographed. In one animal (case 26), the gas score was determined and gas analysis was performed following standardized protocols (19, 20). Representative tissue samples were fixed in 10% neutral buffered formalin, routinely processed, embedded in paraffin, sectioned at thickness of 5 μm, and stained with hematoxylin and eosin for histopathologic analysis.

A conservative approach was adopted to determine fishery interactions, based on previous studies (5–7, 12, 13, 21–24). All stranding cases were reviewed retrospectively, as we looked for individuals with findings that were consistent with fishery interactions, and excluded cases in which other possible traumatic etiologies, such as ship collision, intra-interspecific interactions, or live stranding, could not have been ruled out (25–32). Different types of fishery interactions were determined based on gross findings. Within these various types, histological findings in the skeletal and cardiac muscle, lungs, liver, kidneys, brain, and adrenal glands of the cases with decomposition codes 1–3, were detailed and compared, based on the availability of the samples.

In order to identify factors related to fishery interactions, categorical variables (species, sex, growth development, gonad maturation, body condition, diving behavior, island, and date of stranding) were expressed as frequencies and percentages, and were compared, as appropriate, using the chi-squared (χ^2^) test or the Fisher's exact test. Fishing deaths were due to all of the different types of fishery interactions identified: chronic entanglements, fishermen aggressions from the boat, and bycatch (including fishing net entrapments and ingestion of longline hooks). For statistical analyses, some categorical variables were further regrouped as follows: growth development category (neonate/calf, juvenile/subadult, and adult); body condition (very poor/poor and fair/good); and stranding island based on geographical proximities and the presence of high-site fidelity populations [Western Islands (El Hierro and La Palma), La Gomera together with Tenerife, Gran Canaria, and Eastern Islands (Fuerteventura-Lanzarote-La Graciosa)]. All statistical analyses were performed only on those animals with an identifiable cause of death (453/586). Statistical significance was set at *p* < 0.05. Data were analyzed using the R package, version 3.6.1 (33).

## Results

A total of 860 cetaceans were stranded along the coasts of the Canary Islands between January 2000 and December 2018. Among them, 586 cetaceans were necropsied. The full anatomopathological study of each case allowed us to identify the most probable cause of death in 453 cases. Of those cases, 32 (7.4%) cetaceans of seven species died because of the pathological consequences of fishery interactions.

### Types of Fishery Interactions

Cases of fishery interactions (*n* = 32) were divided into three categories: bycatch (i.e., longline hook ingestion or fishing net entrapment) (*n* = 21); chronic entanglements (*n* = 6); and fisherman aggressions (*n* = 5) (Supplementary Table 1).

#### Bycatch

The bycatch group included cetaceans that were presumably entrapped in active fishing gear. In this group (*n* = 21), four species were affected: the Atlantic spotted dolphin (*n* = 12); striped dolphin (*n* = 6); common dolphin (*n* = 2); and Atlantic bottlenose dolphin (*Tursiops truncatus*) (*n* = 1). All growth development categories were affected by bycatch. The adults were the most affected (11/21), followed by the juveniles (6/21), subadults (3/21), and calves (2/21).

This group was further divided into the following subgroups: dolphins that ingested longline hooks (5/21); dolphins with lesions compatible with forced submersion, which presumably died at depth due to PUE (6/21); dolphins that were stranded alive and later died, exhibiting lesions that were consistent with PUE (3/21); and polytraumatized dolphins that were hauled out alive but suffered additional trauma during handling, including mainly cranioencephalic trauma, and/or perforations produced by fishing equipment (7/21).

**Figure 1:**
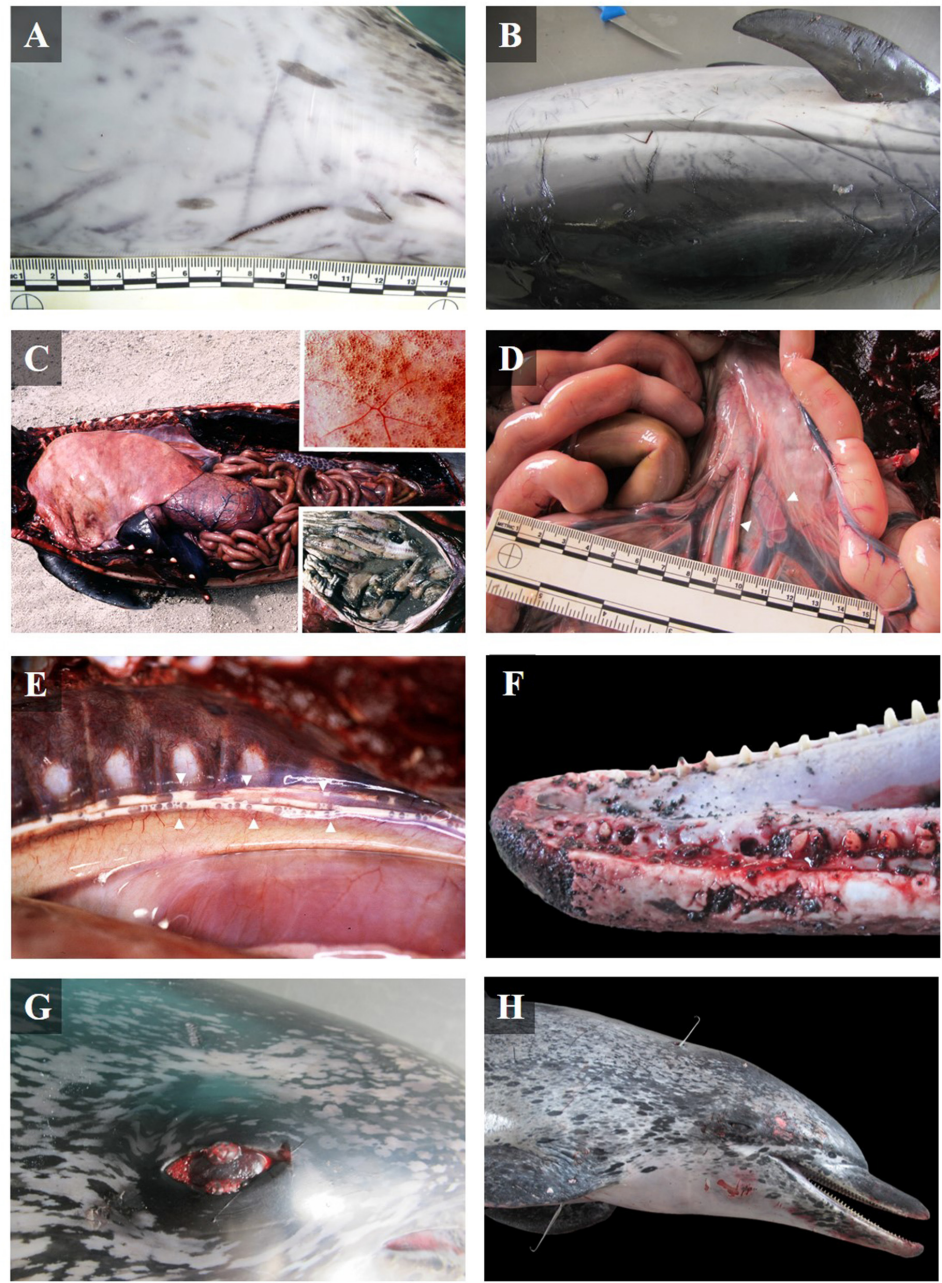
Gross lesions in bycaught dolphins stranded along the Canary Islands. **(A)** Cutaneous impressions presumably produced by a twisted wire net, and **(B)** linear and typical triangular monofilament cuts on an adult Atlantic spotted dolphin (case 29). **(C)** Left lateral view of the thoracic and abdominal cavities of a juvenile Atlantic bottlenose dolphin (case 7). Severe hyperinflated lungs with emphysema (inset upper image), and full stomach with undigested food, fresh fish (inset lower image). **(D)** Mesenteric veins with severe multifocal intravascular gas bubbles in an adult common dolphin (case 32). **(E)** Thoracic duct (between arrow points) full of lymph and gas bubbles in an Atlantic spotted dolphin calf (case 1). **(F)** Left lateral view of the rostrum with multifocal fracture and lost teeth in the rostral part of the mandible (case 21). **(G)** Reddish eyes with conjunctival emphysema of an adult Atlantic spotted dolphin, found with an ingested longline hook (case 29). **(H)** Right lateral view of an adult Atlantic spotted dolphin with two perforating thoracic wounds. The tracks of the wounds, marked by two steel skewers, probably produced during aggressive handling (case 21).

Different degrees of chronicity of the lesions were observed; from acute lesions in PUE cases to subacute-chronic lesions in cases of longline hook ingestion. However, some gross findings were common among most cases (Table 1). Almost every bycaught case showed fair to good body condition. In addition, many animals exhibited superficial cutaneous lesions caused by contact with fishing nets, mainly on the rostrum, but also on the flippers, and along the sides of the body, as well as net impressions (Figures 1A,B). Other common findings included diffuse bilateral hyperinflated lungs (Figure 1C, inset upper image), disseminated intravascular gas bubbles in the veins and lymphatic vessels (Figures 1D,E). In fewer cases, undigested food was found in the forestomach (Figure 1C inset lower image), occasionally in the esophagus, and/or associated with the presence of abundant lymph in the lymphatic mesenteric vessels and chyle in the thoracic duct. Lost teeth (Figure 1F), a fractured rostrum and/or maxilla, and retroperitoneal emphysema were also described. Gross findings of each bycatch case are presented in Supplementary Table 2.

**Table 1:** Gross findings in stranded cetaceans, which died because of fishery interactions (chronic entanglement, aggression, or bycatch) (*n* = 32).

						**Bycatch (n = 21)**									
		**Entanglement (n = 6)**		**Aggression (n = 5)**		**Hook ingestion (n = 5)**		**PUE (n = 6)**		**Aggression during handling (n = 7)**		**Returned (n = 3)**		**Total (n = 21)**	
Stranding event	Stranding alive	1	17%	0	0%	0	0%	0	0%	0	0%	3	100%	3	14%
	Stranding death	5	83%	5	100%	5	100%	6	100%	7	100%	0	0%	18	86%
	Fishing gears attached	4	67%	0	0%	5	100%	0	0%	0	0%	2	67%	7	33%
Bodycondition	Poor-very poor	2	33%	0	0%	0	0%	2	33%	0	0%	0	0%	2	10%
	Good-fair	3	50%	5	100%	5	100%	4	67%	6	86%	3	100%	18	86%
	NE	1	17%	0	0%	0	0%	0	0%	1	14%	0	0%	1	5%
Skin	Net impressions over the body	6	100%	0	0%	2	40%	4	67%	3	43%	1	33%	10	48%
	Net cuts in pectoral flippers	0	0%	0	0%	2	40%	4	67%	2	29%	1	33%	9	43%
	Net cuts in head	1	17%	0	0%	3	60%	5	83%	7	100%	2	67%	17	81%
	Net cuts over the body	1	17%	0	0%	3	60%	3	50%	5	71%	3	100%	14	67%
Subcutaneous	Hematoma	2	33%	5	100%	3	60%	0	0%	6	86%	0	0%	9	43%
Skeletal muscle	Hemorrhages	2	33%	5	100%	2	40%	1	17%	6	86%	0	0%	9	43%
Bones	Mandibles fracture	0	0%	0	0%	2	40%	2	33%	2	29%	2	67%	8	38%
	Maxilla fracture	0	0%	0	0%	1	20%	2	33%	1	14%	2	67%	6	29%
	Neurocranium fracture	0	0%	0	0%	0	0%	0	0%	2	29%	0	0%	2	10%
	Tympanic fracture	0	0%	0	0%	0	0%	0	0%	0	0%	1	33%	1	5%
	Vertebrae fracture	0	0%	2	40%	0	0%	0	0%	1	14%	0	0%	1	5%
	Rib fracture	0	0%	0	0%	0	0%	0	0%	0	0%	1	33%	1	5%
	Teeth lost/fractured	0	0%	2	40%	1	20%	3	50%	3	43%	2	67%	9	43%
Digestive tract	Esophagus Fresh/undigested prey	1	17%	3	60%	0	0%	3	50%	2	29%	0	0%	5	24%
	Stomach Fresh/undigested prey	1	17%	3	60%	2	40%	3	50%	5	71%	0	0%	10	48%
	Digested content	0	0%	0	0%	1	20%	1	17%	0	0%	3	100%	5	24%
	Empty/ND	4	67%	1	20%	2	40%	2	33%	1	14%	0	0%	5	24%
Bubbles	Lymphatic vessels	0	0%	1	20%	0	0%	1	17%	2	29%	0	0%	3	14%
	Blood vessels	1	17%	0	0%	1	20%	5	83%	4	57%	1	33%	11	52%
Blood in cavities	Hemothorax	1	17%	3	60%	2	40%	0	0%	1	14%	0	0%	3	14%
	Hemoabdomen	0	0%	0	0%	3	60%	1	17%	1	14%	0	0%	5	24%
	Hemopericardium	0	0%	0	0%	0	0%	0	0%	1	14%	0	0%	1	5%
Lungs	Hyperinflated	1	17%	0	0%	2	40%	2	33%	5	71%	3	100%	12	57%
	Hemorrhagic parenchyma	0	0%	3	60%	0	0%	1	17%	2	29%	1	33%	4	19%
	Subpleural hemorrhage	0	0%	4	80%	0	0%	3	50%	0	0%	0	0%	3	14%
	Tracheal or bronchial edema	2	33%	0	0%	0	0%	0	0%	2	29%	0	0%	2	10%
	Rib impressions	0	0%	0	0%	0	0%	0	0%	0	0%	2	67%	2	10%
	Rupture of the parenchyma	0	0%	3	60%	0	0%	0	0%	1	14%	0	0%	1	5%
Heart	Hemopericardium	0	0%	0	0%	1	20%	0	0%	0	0%	0	0%	1	5%
	Hemorrhages	0	0%	0	0%	0	0%	0	0%	1	14%	0	0%	1	5%
	Vascular changes in valves	2	33%	0	0%	0	0%	0	0%	0	0%	0	0%	0	0%
Large vessels	Aorta vascular changes	1	17%	0	0%	1	20%	1	17%	4	57%	0	0%	6	29%
	Rete mirabile vascular changes	0	0%	0	0%	0	0%	0	0%	2	29%	0	0%	2	10%
Kidney	Hemorrhages	0	0%	0	0%	0	0%	0	0%	1	14%	0	0%	1	5%
	Retroperitoneal emphysema	1	17%	0	0%	1	20%	2	33%	2	29%	0	0%	5	24%
Lymphatic	Lymph in vessels	0	0%	2	40%	0	0%	3	50%	4	57%	0	0%	7	33%
Brain	Meningeal hemorrhages	0	0%	1	20%	0	0%	0	0%	3	43%	0	0%	3	14%
	Parenchymal hemorrhages	0	0%	0	0%	0	0%	0	0%	1	14%	0	0%	1	5%

*Decomposition codes (1–5). For each category, the number and percentage of affected individuals are shown*.

*The color values correspond to the percentage of a finding or a pathologic feature within each group of studied animals (bycatch, fisherman aggression and chronic entanglement)*.

Histologically, almost all cases showed clear intravascular spaces compatible with gas and/or fat embolism. Common findings included: mild to moderate multifocal acute degenerative changes in skeletal muscle (i.e., segmental degeneration of the muscular fibers); mild multifocal acute degenerative changes in cardiac muscle (i.e., increased acidophilic cytoplasm of the myocardiocytes, contraction band necrosis, and juxtanuclear vacuolization of cardiac cells); multifocal lung changes such as alveolar emphysema, hemorrhages, and alveolar edema; systemic leukocytosis; and multifocal intracytoplasmic hepatocellular hyaline globules. In fewer cases, bronchiolar sphincter contraction, multifocal hyaline casts in distal renal tubules and corticomedullary adrenal hemorrhages, intramuscular hemorrhages, and multifocal pigmentary tubulonephrosis were observed. Regarding the central nervous system (CNS), mild changes manifested as multifocal hemorrhages, perivascular edema, and perivascular cuffs mostly associated with glial nodules were observed in a few cases. A severe non-suppurative meningoencephalitis was present in one case (Table 2). Histological findings of each bycatch case are presented in Supplementary Table 3.

**Table 2:** Histological findings in stranded cetaceans, which died because of fishery interactions.

							**Bycatch**									
			**Entanglement**		**Aggression**		**Hook ingestion**		**PUE**		**Aggression during handling**		**Returned**		**Total**	
Skeletal muscle	Acute degenerative changes	No	1/5	20%	1/4	25%	1/3	33%	0/5	0%	0/6	0%	0/3	0%	1/17	6%
		Mild	3/5	60%	1/4	25%	1/3	33%	3/5	60%	2/6	33%	3/3	100%	9/17	53%
		Moderate	1/5	20%	2/4	50%	1/3	33%	1/5	20%	3/6	50%	0/3	0%	5/17	29%
		Severe	0/5	0%	0/4	0%	0/3	0%	1/5	20%	1/6	17%	0/3	0%	2/17	12%
	Atrophy		3/5	60%	1/4	25%	0/3	0%	1/5	20%	2/6	33%	0/3	0%	3/17	18%
	Hemorrhages		0/5	0%	1/4	25%	1/3	33%	0/5	0%	4/6	67%	0/3	0%	5/17	29%
Lungs	Alveolar edema		2/5	40%	4/5	80%	3/3	100%	4/6	67%	4/6	67%	0/2	0%	11/17	65%
	Emphysema		3/5	60%	3/5	60%	2/3	67%	5/6	83%	4/6	67%	2/2	100%	13/17	76%
	Muscular bronchiolar sphincter contraction		0/5	0%	4/5	80%	2/3	67%	2/6	33%	2/6	33%	0/2	0%	6/17	35%
	Hemorrhages		1/5	20%	4/5	80%	2/3	67%	4/6	67%	4/6	675	2/2	100%	12/17	71%
Heart	Acute degenerative changes	No	0/5	0%	0/4	0%	0/3	0%	1/5	20%	4/6	67%	1/1	100%	6/15	40%
		Mild	5/5	100%	1/4	25%	1/3	33%	4/5	80%	1/6	17%	0/1	0%	6/15	40%
		Moderate	0/5	0%	0/4	0%	2/3	67%	0/5	0%	1/6	17%	0/1	0%	3/15	20%
		Severe	0/5	0%	0/4	0%	0/3	0%	0/5	0%	0/6	0%	0/1	0%	0/15	0%
	Hemorrhages		2/5	40%	0/4	0%	0/3	0%	0/5	0%	3/6	50%	0/1	0%	3/15	20%
Liver	Intracytoplasmic hyaline globules		4/5	80%	2/3	67%	2/3	67%	4/4	100%	4/5	80%	1/3	33%	11/15	73%
Adrenal glands	Hemorrhages		4/5	80%	2/4	50%	1/3	33%	2/4	50%	1/6	17%	1/3	33%	5/16	31%
Blood vessels	Leukocytosis		4/5	80%	3/5	60%	3/3	100%	5/6	83%	5/6	83%	2/3	67%	15/18	83%
	Intravascular coagulation		2/5	40%	0/5	0%	1/3	33%	1/6	17%	5/6	83%	0/3	0%	7/18	39%
	Intravascular clear spaces		5/5	100%	5/5	100%	3/3	100%	6/6	100%	5/6	83%	3/3	100%	17/18	94%
Reproductive system	Gonadal maturity	Mature	2/5	40%	3/5	60%	3/3	100%	2/6	33%	5/6	83%	1/3	33%	11/18	61%
		Immature	3/5	60%	2/5	40%	0/3	0%	4/6	67%	1/6	17%	2/3	67%	7/18	39%
Kidney	Membranous glomerulonephritis		0/5	0%	2/5	40%	1/3	33%	1/6	17%	1/6	17%	0/3	0%	3/18	17%
	Hyaline cast		1/5	20%	0/5	0%	2/3	67%	1/6	17%	2/6	33%	0/3	0%	5/18	28%
	Pigmentary tubulonephrosis		0/5	0%	1/5	20%	0/3	0%	2/6	33%	1/6	17%	1/3	33%	4/18	22%
	Hemorrhages		2/5	40%	0/5	0%	0/3	0%	0/6	0%	1/6	17%	0/3	0%	1/18	6%
Brain	Perivascular edema		1/4	20%	3/4	75%	1/3	33%	1/6	17%	2/6	33%	1/3	33%	5/18	28%
	Hemorrhages		2/4	50%	1/4	25%	2/3	67%	2/6	33%	3/6	50%	1/3	33%	8/18	44%
	Meningitis/encephalitis		1/4	25%	1/4	25%	0/3	0%	0/6	0%	0/6	0%	1/3	33%	1/18	6%
	Perivascular cuffs		0/4	0%	2/4	50%	0/3	0%	1/6	17%	3/6	50%	0/3	0%	4/18	22%
	Glial nodules		3/4	75%	0/4	0%	1/3	33%	1/6	17%	2/6	33%	0/3	0%	4/18	22%

*For cases with decomposition codes 1–3 (n = 28), histological findings in the skeletal and cardiac muscle, lungs, liver, kidneys, adrenal glands, blood vessels, and brain, were detailed and compared, as well as findings of gonad maturation (M, mature; I, immature) upon the availability of samples*.

*The color values correspond to the percentage of a finding or a pathologic feature within each group of studied animals (bycatch, fisherman aggression and chronic entanglement)*.

**Figure 2:**
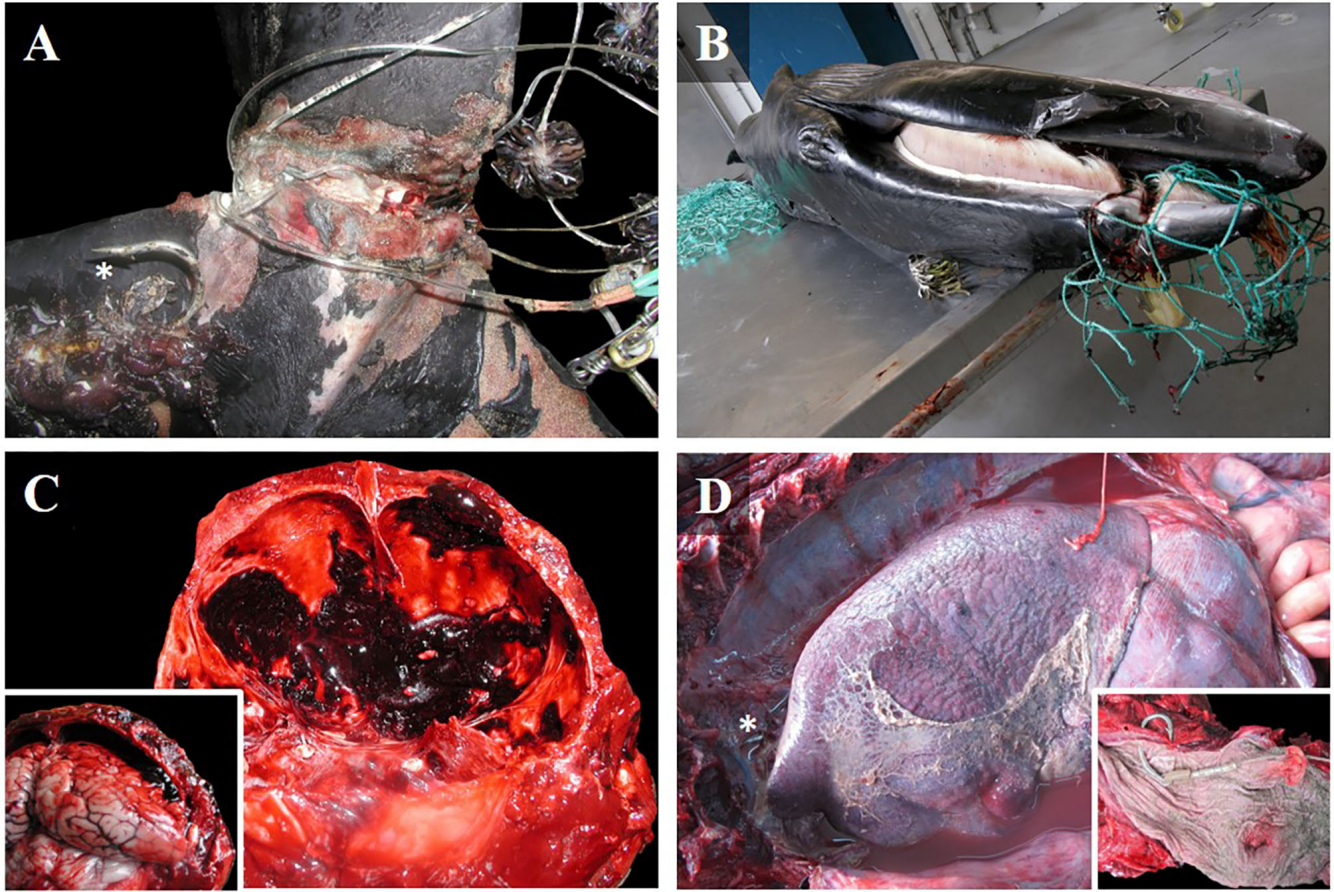
Gross findings in cetacean deaths caused by different types of fishing interactions. **(A)** Dorsal view of the caudal peduncle of a Gervais' beaked whale calf (case 10), entangled in a longline. Cirripeds and a hook (asterisk) were attached to the nylon wire. Severe chronic skin ulcerations were observed, with a loss of soft tissue, and granulation tissue on the borders of the wound. Note that the fluke was almost amputated. **(B)** Lateral view of entangled minke whale calf (case 16) with chronic ulcerative stomatitis affecting the maxilla, and tissue strangulation. **(C)** Caudal view of the cranium of an adult Atlantic spotted dolphin (case 13) with a fatal subdural hematoma, and associated brain compression (inset). **(D)** Left lateral view of the thoracic cavity of an adult Atlantic spotted dolphin (case 29). Fibrinosuppurative pleuritis and pyothorax caused by ingestion of a longline hook (asterisk) that perforated the thoracic portion of the esophagus (inset).

All animals affected by the ingestion of longline hooks (*n* = 5) were adult Atlantic spotted dolphins in fair-good body condition. Ingested hooks had perforated the esophagus (cases 2 and 29), produced fibrinosuppurative pleuritis and pericardial hemorrhages (case 29; Figure 2D), pierced the mandibular fossa (cases 18 and 31), and affected the sublingual soft tissue to produce focal, extensive necrosis and hemorrhage (case 20). Hemothorax, hemoabdomen, and hemopericardium were also observed (Table 1). In one case, reddened eyes were observed (Figure 1G).

Of the six cases showing findings consistent with PUE, four were striped dolphins [one pregnant adult (case 17), two subadults (cases 28 and 30), and one juvenile (case 25)]; one was a Atlantic spotted dolphin calf (case 1); and another, a juvenile Atlantic bottlenose dolphin (case 7). All animals in this category were found dead, and the majority were in good-fair body condition. All had cutaneous lesions associated with contact with fishing nets, as well as disseminated intravascular gas bubbles, cutaneous impressions, lost/fractured teeth, undigested food, hyperinflated lungs with subpleural hemorrhages and hemorrhagic parenchyma, and retroperitoneal emphysema (Table 1).

Three other animals showed lesions compatible with PUE. They were likely caught in nets and released alive back into the ocean. They included a striped dolphin calf (case 6), a juvenile striped dolphin entangled in gear with a fishing ball (case 12), and an adult Atlantic spotted dolphin that appeared to have been entangled in a fishing net (case 23). Supposedly, shortly after release, these animals were stranded alive and subsequently died. Two had fractured bones [mandible and maxilla with lost or fractured teeth, tympanic fracture, and rib fracture], which may have been caused by active stranding (Table 1). Disseminated intravascular gas bubbles were present in case 23 alone. The same animal had bubbles within the posterior chamber of the eye.

Bycaught cases included seven animals that were probably hauled out and raised to the deck alive but suffered different forms of physical trauma during handling. The affected cases included Atlantic spotted dolphins (*n* = 5) [three adults (cases 11, 21, and 22); one subadult (case 27); and one juvenile (case 9)], and common dolphins (*n* = 2) [one adult (case 32) and one juvenile (case 26)]. All were stranded dead, and most were in fair body condition. These animals were polytraumatized and exhibited skin-muscle perforations (Figure 1H) with associated hemorrhages, which affected internal organs in some cases [perforations of the aorta (case 9), trachea and esophagus (case 22)]; neurocranium fractures (2/7) [in the squamous part of the occipital bone (case 27), and in the right occipital condyle (case 32)] with associated hematoma and congestion in the underlying leptomeninges and brain; and fractures of the maxilla and mandible (cases 26 and 32). Case 26 also showed signs of severe trauma to the right caudolateral side of the head with leptomeningeal congestion, severe scoliosis of the peduncle, and an open fracture that affected the caudal vertebrae. Other traumatic findings included hemorrhages on the adventitia of the aorta (cases 9, 11, 22, and 26) and in the rete mirabile (cases 22 and 26); hemothorax (cases 11 and 22); hemoabdomen (case 22); hemopericardium (case 22); and lung perforation (case 11) (Table 1).

Disseminated intravascular gas bubbles were present in four out of seven bycaught animals. One was a juvenile female common dolphin (case 26) that was found stranded dead, refrigerated (4°C) for 24 h, and necropsied while still fresh (decomposition code 2). The gas score and gas analyses were evaluated on this animal. The gas score revealed the presence of occasional small bubbles following careful screening of the subcutaneous veins (gas score 1); abundant presence of gas bubbles in the coronary veins and lumbocaudal venous plexus (gas score 5); and gas bubbles occupying complete sections of the mesenteric veins (gas score 6). Emphysema was present exclusively in the perirenal subcapsular region.

**Figure 3:**
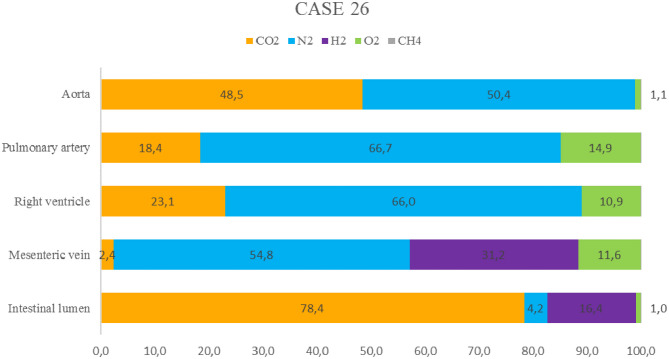
Relative gas composition (% μmol) of samples from the intestine, mesenteric veins, right ventricle, aorta, and pulmonary artery of a juvenile female common dolphin (case 26).

Gas analyses were performed in mesenteric veins, the right ventricle, aorta, pulmonary artery, and intestinal lumen (Figure 3). Except for samples of the intestine and mesenteric veins, N_2_ was the main component of the sampled bubbles [61.0 ± 9.2 μmol %], followed by CO_2_ [30.0 ± 16.2 μmol %], and O_2_ [8.9 ± 7.1 μmol %]. Both CH_4_ and H_2_ were absent from these samples. In contrast, gas bubbles from the mesenteric veins contained H_2_ [31.2 ± 10.2 μmol %], in addition to N_2_ [54.8 ± 3.7 μmol %], O_2_ [11.6 ± 3.2 μmol %], and CO_2_ [2.4 ± 3.3 μmol %]. In the intestinal lumen, CO_2_ was the main constituent [78.4 ± 2 μmol %], followed by H_2_ [16.4 ± 1.3 μmol %], N_2_ [4.2 ± 2.6 μmol %], and O_2_ [1 ± 0.5 μmol %].

#### Chronic Entanglement

In this category, we observed six cases with skin wounds that were consistent with chronic entanglement. They included two minke whale calves (*Balaenoptera acutorostrata*) (cases 16 and 19); two short-finned pilot whale calves (*Globicephala macrorhynchus*) (cases 14 and 24); one Gervais' beaked whale calf (*Mesoplodon europaeus*) (case 10); and one subadult Atlantic bottlenose dolphin (case 8). Regarding body condition, three out of six cases showed fair to good body condition (cases 8, 14, and 19), while two were in poor-very poor condition (cases 10 and 16). In case 24, the advanced decomposition (code 5) did not allow us to determine the body condition, nor perform histological evaluation.

All cases exhibited fishing gear impressions, erosions, and/or ulcerative lesions with granulation tissue and fibrosis over the rostrum, flippers, and/or tail. In case 14, the animal was stranded alive with nylon wire attached to a plastic floating bottle. The other five were found dead. Case 8 exhibited rope impressions between the pectoral fins and near the left eye. In case 10, nylon wire with cirripeds and longline hooks were attached to the caudal peduncle (Figure 2A). Case 16 had green monofilament nylon fishing gear attached to the maxilla (Figure 2B). Case 19 exhibited symmetrical bilateral ulcers and granulation tissue in the mandibular symphysis, consistent with fishing gear impressions. Case 24 had a thick rope (4 cm in diameter) surrounding the thoracic region. Other pathological findings included tracheal edema, hyperinflated lungs with rib impressions, serous atrophy of pericardial fat, hemorrhage in the adventitia of the thoracic aorta, and retroperitoneal emphysema. Only two cases had undigested food in the stomach (Table 1). Gross findings of each case are presented in Supplementary Table 2.

Histologically, clear intravascular spaces, compatible with intravascular gas and/or fat, mild to moderate multifocal acute muscular degenerative changes in skeletal muscle, and mild degenerative changes in the cardiac muscle, multifocal myofiber atrophy of the skeletal muscle, multifocal corticomedullary adrenal hemorrhages, intracytoplasmic hepatocellular hyaline globules, systemic leukocytosis, hemorrhages in multiple organs, intravascular coagulation, multifocal alveolar emphysema, alveolar edema, diffuse hemorrhagic lung parenchyma, multifocal hemorrhages in the cortex of the kidney, and hyaline casts were observed. Regarding the CNS, case 8 showed mild focal non-suppurative meningoencephalitis. Mild multifocal glial nodules and moderate multifocal hemorrhages were occasionally observed (Table 2). Histological findings are presented in Supplementary Table 3.

#### Fisherman Aggression

In this category (*n* = 5), we observed three Atlantic spotted dolphins [two adults (cases 3 and 13) and one juvenile (case 15)], and two common dolphins [one adult and one calf, possibly relatives as they were stranded on the same date and location (cases 4 and 5)]. All cetaceans in this group showed fair-good body condition, and a full stomach with undigested prey, as well as signs of anthropogenic trauma, such as unique or multifocal stabs inflicted by sharp instruments, mainly in the laterodorsal area affecting the thoracic region (cases 3, 4, 5, and 15) and dorsal side of the head (cases 3 and 13). Net cuts and impressions were not observed on the skin (Table 1). These animals exhibited several lesions associated with incisive trauma, such as vascular changes (i.e., edema, hemorrhage, hematoma) in the skin and muscular tissue, bone fractures [sixth left rib (case 4) and the ninth thoracic vertebrae (case 5)], lung perforations with related focal, extensive hemorrhage, and hemothorax (cases 4, 5, and 15), and hemorrhage in the leptomeninges (case 13; Figure 2C) were observed (Table 1). Gross findings of each case are presented in Supplementary Table 2.

Histologically, mild to moderate multifocal acute segmental myofiber degeneration of skeletal muscle, diffuse hemorrhages within the affected skin and muscles, mild multifocal acute degenerative changes in cardiac muscle, multifocal alveolar edema, hemorrhages in the lung parenchyma, muscular sphincter contraction of the bronchioles, multifocal emphysema, systemic leukocytosis, intracytoplasmic hepatocellular hyaline globules, multifocal corticomedullary adrenal gland hemorrhages, multifocal membranous glomerulonephritis, and multifocal pigmentary tubulonephrosis were observed. Regarding the CNS, multifocal perivascular edema, perivascular cuffs, and hemorrhages in the brain parenchyma were occasionally observed. Mild multifocal granulomatous encephalitis was present in case 3. Clear intravascular spaces were present in all cases (Table 2). Histological findings are presented in Supplementary Table 3.

### Statistical Analysis

Although almost half of the affected animals were Atlantic spotted dolphins [46.9% (15/32)], no statistically significant differences were found in the prevalence of fishery interactions among animals of different species (*p* = 0.125), nor among growth development categories (*p* = 0.871), sex (*p* = 0.813), gonad maturation (*p* = 0.704), or the island of stranding (*p* = 0.684) (Table 3).

**Table 3:** Statistical analysis of the epidemiological data of studied cetaceans during the period 2000–2018 (n = 586).

	**Overall N = 586**	**Other cause of death N = 421**	**Fishing interactions *N* = 32**	**P-value**
**Species**				0.125
*Balaenoptera acutorostrata*	6 (1.3)	4 (1.0)	2 (6.2)	
*Balaenoptera borealis*	2 (0.4)	2 (0.5)	0 (0.0)	
*Balaenoptera edeni*	2 (0.4)	2 (0.5)	0 (0.0)	
*Balaenoptera physalus*	4 (0.9)	4 (1.0)	0 (0.0)	
*Delphinus delphis*	51 (11.3)	47 (11.2)	4 (12.5)	
*Globicephala macrorhynchus*	36 (7.9)	34 (8.1)	2 (6.2)	
*Grampus griseus*	11 (2.4)	11 (2.6)	0 (0.0)	
*Kogia breviceps*	23 (5.1)	23 (5.5)	0 (0.0)	
*Kogia sima*	6 (1.3)	6 (1.4)	0 (0.0)	
*Lagenodelphis hosei*	3 (0.7)	3 (0.7)	0 (0.0)	
*Megaptera novaeangliae*	2 (0.4)	2 (0.5)	0 (0.0)	
*Mesoplodon bidens*	1 (0.2)	1 (0.2)	0 (0.0)	
*Mesoplodon densirostris*	7 (1.5)	7 (1.7)	0 (0.0)	
*Mesoplodon europaeus*	6 (1.3)	5 (1.2)	1 (3.1)	
*Orcinus orca*	1 (0.2)	1 (0.2)	0 (0.0)	
*Phocoena phocoena*	1 (0.2)	1 (0.2)	0 (0.0)	
*Physeter macrocephalus*	22 (4.9)	22 (5.2)	0 (0.0)	
*Pseudorca crassidens*	2 (0.4)	2 (0.5)	0 (0.0)	
*Stenella coeruleoalba*	92 (20.3)	86 (20.4)	6 (18.8)	
*Stenella frontalis*	89 (19.6)	74 (17.6)	15 (46.9)	
*Stenella longirostris*	3 (0.7)	3 (0.7)	0 (0.0)	
*Steno bredanensis*	23 (5.1)	23 (5.5)	0 (0.0)	
*Tursiops truncatus*	36 (7.9)	34 (8.1)	2 (6.2)	
*Ziphius cavirostris*	24 (5.3)	24 (5.7)	0 (0.0)	
**Sex**				0.813
Female	205 (45.8)	191 (45.9)	14 (43.8)	
Male	243 (54.2)	225 (54.1)	18 (56.2)	
**Growth development categories**				0.871
Neonate/calf	131 (28.9)	123 (29.2)	8 (25.0)	
Juvenile/subadult	130 (28.7)	120 (28.5)	10 (31.2)	
Adult	192 (42.4)	178 (42.3)	14 (43.8)	
**Coast**				0.684
El Hierro y La Palma	8 (1.8)	7 (1.7)	1 (3.1)	
La Gomera y Tenerife	154 (34.0)	141 (33.5)	13 (40.6)	
Gran Canaria	125 (27.6)	116 (27.6)	9 (28.1)	
Fuerteventura y Lanzarote	166 (36.6)	157 (37.3)	9 (28.1)	
**Mature categories**				0.704
Immature	225 (50.1)	210 (50.4)	15 (46.9)	
Mature	224 (49.9)	207 (49.6)	17 (53.1)	
**Body condition**				0.004
Poor/very poor	162 (37.8)	158 (39.6)	4 (13.3)	
Good/fair	267 (62.2)	241 (60.4)	26 (86.7)	
**Diving behavior**				0.008
Shallow diver	317 (70.0)	288 (68.4)	29 (90.6)	
Deep diver	136 (30.0)	133 (31.6)	3 (9.4)	

*The pathological cause of death in 453 cases was determined, among which, 32 cases were due to fishery interactions*.

#### Body Condition

Body condition could have been determined in 94.7% of the individuals, with a known cause of death (429/453). Most of the animals [62.2% (267/429)] showed good/fair body condition, while 37.8% (162/429) were in poor/very poor body condition. The body condition of two dolphins, which died as a result of fishery interactions, could not have been determined owing to artifactual loss of tissue (case 9) and the very advanced state of decomposition of the carcass (code 5) (case 24) (Supplementary Table 1). Nonetheless, most dolphins showing signs of fishery interactions were in good/fair body condition 86.7% (26/30), compared with dolphins in poor/very poor condition. This difference was statistically significant (*p* = 0.004) (Table 3).

#### Diving Behavior

Shallow-water species were stranded in greater numbers, representing 70% of the animals with a known cause of death (317/453), while deep divers represented 30% of the cases (136/453). Although we observed more shallow than deep divers stranded during the study period, this difference was even larger when the prevalence of shallow [90.6% (29/32)] vs. deep divers [9.4% (3/32)] was compared with the findings of fishery interactions. This difference was statistically significant (*p* = 0.008) (Table 3).

#### Temporality of Stranding Events

The yearly average number of stranding caused by fishery interactions was 1.7 animals (32 cases over 19 years), indicating a low rate of fishery interactions within the geographical area. In 2001 and 2017, a slight increase in the number of cases (*n* = 5 each year) was noted. No fishery interactions were recorded during the years 2003, 2006, 2010, or 2011.

## Discussion

The rates of fishery interactions among cetaceans are underreported worldwide (2, 3). In the case of stranded cetaceans, an advanced decomposition code may hinder the determination of the cause of death. In addition, most fishery interaction findings (except gear cuts and impressions) are not pathognomonic (4, 5). Nonetheless, fisheries are considered a major global threat to cetaceans (34). Bycatch especially affects the harbor porpoise (*Phocoena phocoena*), bottlenose dolphin, common dolphin, and striped dolphin [e.g., (1, 35, 36)]. Furthermore, cases of chronic entanglements appear to be on the rise globally (8, 10), and their effects in certain baleen populations such as the minke whale is concerning (34).

In the Canary Islands, fishing is mainly artisanal and multi-specific, and is characterized by the use of small vessels (≤15 m in total length) and various types of fishing gear. Larger vessels are also used for tuna, but to a lesser extent^2^. Gillnets are allowed during certain periods in some designated areas, whereas trawling is absolutely forbidden in the archipelago. In addition, longlines and traps are forbidden in El Hierro and Fuerteventura, as well as in all marine reserves of the archipelago [Annex 1. Decreto 182/2004 de 21 de diciembre, Reglamento de la Ley de Pesca de Canarias]. These conservative policies may explain the low annual rates of fishing-related deaths in cetaceans stranded along the Canary coasts.

Of the seven cetacean species identified, the Atlantic spotted dolphin was the most affected. This species, together with the Atlantic bottlenose dolphin, Gervais' beaked whale, short-finned pilot whale, and striped dolphin are regularly present year-round. Moreover, common dolphins and minke whales are seasonally present (37, 38).

### Bycatch

Fishery interactions were likely recent and occurred close to the Canary coast in animals that were stranded alive or found dead in a fresh or very fresh state. Although local artisanal fisheries might have been responsible for these deaths, other fishing activities in international waters and illegal fishing cannot be ruled out. Until now, no pathognomonic clinical findings have been identified for bycaught animals (4). However, the greater the number of compatible lesions identified in a case, the more consistent the identification of bycatch (5, 39).

In this study, the ingestion of longline hooks affected Atlantic spotted dolphins alone (*n* = 5). Although this interaction with longline fisheries is well-known worldwide (40, 41), its related pathologies have been poorly reported. In this study, we identified two cases of Atlantic spotted dolphins with hooks that pierced the mandibles. To the best of our knowledge, this is also the first report of esophageal perforation with fibrinosuppurative pleuritis caused by hook ingestion in a dolphin species (Figure 2D). Although the literature reflects more information about larynx strangulation with longline fishing gear (42, 43), this type of lesions was not observed in the present study.

Different necropsy findings might be observed in cases of fishing net entrapment, depending on the fishery and the affected cetacean species (5, 21, 22, 44). Our results are consistent with those of Bernaldo de Quirós et al. (6), who reported external net marks, evidence of recent feeding (fresh undigested gastric contents), and disseminated intravascular gas bubbles.

In contrast with the findings of Bernaldo de Quirós et al. (6), only one case had reddish eyes (case 29), and another case had bubbles within the posterior chamber of the eye (case 23). In the present study, hyperinflated and hemorrhagic lungs were also identified, with occasional froth and rib impressions, corresponding histologically with areas of marked emphysema and alveolar edema. These findings agreed with those of Moore et al. (5). However, Bernaldo de Quirós et al. (6) found that froth in the airways and other lung changes (i.e., wet, heavy edema, congestion, and hemorrhage) were statistically poor indicators of bycatch.

In the present study, case 26 was one example of a bycaught dolphin with gas embolism. This animal exhibited a large number of gas bubbles (gas score of 18), consistent with the findings of Bernaldo de Quirós et al. (24). In addition, the composition of the gas bubbles was consistent with gases produced by compression and decompression, in which nitrogen is the main component, and CO_2_ can sometimes be present at high concentrations (20). Hydrogen, a marker of putrefaction, was found in the mesenteric veins alone, which is consistent with the findings of Bernaldo de Quirós et al. (24).

Pathological findings in cases of fishing net entrapment often suggest some degree of physical struggle associated with varying degrees of muscular exertion (5). In bycaught dolphins, the adrenocortical response and hyperthermia (45) induce injury to the skeletal muscle, similar to that described in live stranded cetaceans (26, 27, 31). Acute degenerative changes have also been observed in severely polytraumatized free-ranging stranded cetaceans, such as in cases of ship strikes (23, 28) and fatal social traumatic intra-interspecific interactions (23, 32). Previous studies have reported cardiac changes (29, 46, 47), as well as intracytoplasmic hepatocellular hyaline globules (13, 27, 48) in agonal situations. Intravascular coagulation, mostly present in bycaught dolphins that endured aggression during handling, has been described in domestic animals with extensive tissue destruction (49). We also observed a few cases with mild inflammation of the CNS. These findings are consistent with the presence of concomitant infections.

### Chronic Entanglement

It is difficult to know the actual number of entangled cetaceans even if the number of entangled individuals within a specific population is known, as the same animal can become entangled multiple times (50), and entangled carcasses tend to sink (8). Even with the limitations of the available data, the number of reported entanglements during the last decade is three times higher than that reported during the 1990s, and 97% of those cases were entangled in fishing gear (10).

In the Atlantic Ocean, at least half of the mysticetes' deaths are caused by fishing gear (51). Minke whales particularly, appear to be less likely to survive entanglements than larger whales (52). The first description of an entangled minke whale in the Canary Islands was reported in 1993 in Morro Jable-Fuerteventura. This stranded animal was found dead, with a fishing net in the rostrum (53). In recent years (2012–2020), entanglements have included seven minke whales, six Bryde's whales (*B. edeni*), one humpback whale (*Megaptera novaeangliae*)^3^, two rough-toothed dolphins (*Steno bredanensis*), and one member of the Delphinidae family, sighted close to the Canary coasts (personal communication with the Canary Islands stranding network). Among these, three minke whales and two Bryde's whales were disentangled^4^. Whether those whales survived remains unknown, as no follow-up investigations were conducted after the disentanglement. Consistent with the findings of the present study, we consider chronic entanglements a potential threat to cetacean' populations in the Canary Islands, especially minke and Bryde's whales.

Entangled mysticetes are commonly found with ropes and nets within the oral cavity, or surrounding the flippers, or tail, and entangled odontocetes are usually found with recreational fishing gear, longlines, and fishing lures (5). Even if the gear becomes detached, the scars of healed wounds may remain (54). In chronic entanglements, open and unhealed wounds could lead to septicemia and even death (5). Although no microbiological studies have been conducted on entangled cases in the present study, histological evaluation of the cases revealed intravascular coagulation, leukocytosis (mostly neutrophilic), and multiorgan hemorrhages in most of the affected animals.

In addition, those cases in poor body condition showed atrophy of the skeletal muscle. Case 19 also exhibited severe serous atrophy of pericardial fat, which indicates a catabolic condition. In the present study, only one animal was stranded alive and most cases showed mild degenerative changes in myocardiocytes. The occurrence of cardiac failure in cases of chronic entanglement should be further investigated. Similarly, the presence of hepatocellular hyaline globules in almost all cases was remarkable, previously described in agonal situations of stranded cetaceans (27, 48).

### Fisherman Aggression

The predominance of artisanal fisheries in the Canary Islands suggests daily direct contact between cetaceans and fishermen, who are usually in small vessels a few meters above. Our results showed lethal trauma on the dorsal side of the dolphins, which is consistent with the fishermen's position just above, on the water's surface. Stabs and contusions affected mainly the thoracic cavity (with associated hemothorax, bone fractures, lung perforations), and/or the cephalic region (head contusions with associated brain hemorrhages). No other common findings were observed between these cases and the bycatch cases (e.g., net cuts/impressions on the skin, hyperinflated lungs, intravascular disseminated gas bubbles), nor evidence of being brought to deck. Although this category has been previously described, few cases have been reported (7, 55), because the most frequent aggressions inflicted by fishermen (amputations, stabs, and perforations) occur onboard, when animals are trapped in gillnets or trawlnets (5). Another form of human aggression is gunshot wounds (56), which were not observed in the present study. Histological findings, such as severe focal, extensive hemorrhages in the skin and muscles, and hemorrhagic lungs, agreed with the gross findings, which indicated that the various cases of trauma were inflicted while the animals were still alive. Histological findings were associated with agonal perimortem changes in dolphins, as well as systemic leukocytosis, due to open wounds.

### Statistical Analysis

#### Body Condition

The body condition index is a good indirect indicator of the nutritional status of cetaceans (17). Previous studies have described bycaught dolphins in good body condition (4), but this is common in cetacean deaths due to the fatal trauma of ship strikes (5, 7, 12, 13), and social traumatic intra-interspecific interactions (32).

Our results indicate that individuals with good nutritional status may be more susceptible to adverse fishery interactions (especially bycatch), compared with animals exhibiting catabolic metabolism due to energy demanding pathologies (i.e., severe infections, parasitism, or neoplasia). In contrast, chronic entangled cetaceans typically appear in poor body condition (5, 7). Our results showed only a few necropsied cases of entanglement, only half of which showed poor-very poor body condition. However, observational records along the Canary coasts have confirmed the presence of entangled whales in poor body condition (personal communication with the Canary Islands stranding network).

#### Diving Behavior

In the present study, shallow diving species were affected by fishery interactions to a significantly greater extent than deep diving species. These findings are consistent with those of previous studies, in which the majority of the affected cetaceans were shallow-water species (5, 10, 40). In the Canary Islands, artisanal fishing activities are mainly pelagic and superficial (0–150 m depth)^5^, which is consistent with the swimming patterns of shallow species.

Nonetheless, it is important to highlight the fact that deep divers, especially sperm whales (*Physeter macrocephalus*), have been reported worldwide with considerable amounts of ingested marine debris, including fishing gear (10, 57, 58). The Canary waters contain a great variety of deep divers, which are known to be affected by marine debris (59). We highly recommend more detailed descriptions of ingested foreign bodies, to better understand whether they originate from fishery activities.

#### Temporality of Stranding Events

Fortunately, our geographical area showed relatively low numbers of deaths due to fishing activities. This might be related to the sustainable artisanal fisheries and restrictive laws in the Canary waters. Similarly, in the Azores of Portugal, bycatch rates are low, with no evidence of any increase over the last 15 years (60). Artisanal fisheries cause a considerable number of deaths worldwide, even though these numbers are typically lower than those caused by industrial fisheries (1, 61).

In recent years, although sightings of live chronic entangled cetaceans have increased along the archipelago, necropsied cases remain scarce. The widespread use of social media to report the consequences of pollution in marine ecosystems has helped us to learn more about these interactions.

## Conclusions

This is the first retrospective study of fishery interactions in stranded cetaceans along the Canary Islands. The determination of different types of fishery interactions as the cause of death was based on a 20-year investigation of stranded cetaceans. We described the most relevant gross and histologic findings in each type of interaction. Three types of fishery interactions were observed according to gross findings, including chronic entanglement, fisherman aggression, and bycatch (longline hook ingestion and fishing net entrapment).

We found that the Atlantic spotted dolphin, regularly seen in our geographical area, was the most affected species. We also found that cetaceans in good-fair body condition and shallow-water species were significantly more affected. The low prevalence of fishery interactions in this region could be attributed to the broad protective legislation applicable to the Canary waters and the most prevalent type of fisheries, artisanal fisheries. However, there is increasing concern about chronic entanglements, especially in minke and Bryde's whales. We encourage continuous pathological studies on stranded cetaceans, to monitor fishing-related deaths and their consequences in cetacean populations. The findings of this study might contribute to the implementation of appropriate conservation policies in the Canary Islands.

## Data Availability Statement

All datasets generated for this study are included in the article/Supplementary Material.

## Author Contributions

RP-L, YB, MArb, AF, and ES: conceptualization. RP-L, AF, ES, JD, JD-D, SS, A, NG-Á, DZ, AX, MR, IF-J, FC, PD-S, SS-G, NC, CS-S, MArr, YB, and MArb: sampling and diagnosis of the cause of death of each animal. PS, RP-L, and YB: data analyses. RP-L and MR: image editing. RP-L: writing. YB, MArb, MR, AF, and ES: supervision. All authors: review and editing.

## Conflict of Interest

The authors declare that the research was conducted in the absence of any commercial or financial relationships that could be construed as a potential conflict of interest.

## Footnotes

^1^https://www.biodiversidadcanarias.es/

^2^https://www.gobiernodecanarias.org/pesca/

^3^https://twitter.com/GranCanariaCab/status/1209437122345734145

^4^https://www.eldiario.es/canariasahora/sociedad/Liberada-canarias-ballena-atrapada-marana_0_76292969.html

^5^http://www.grancanariapescaenred.com/sobre-nosotros/como-pescamos/
