# Supplementary material for: Retrospective Study of Fishery Interactions in Stranded Cetaceans, Canary Islands
Source: Front Vet Sci. 2020 Oct 21;7:567258. doi: 10.3389/fvets.2020.567258 (PMC7641611; doi:10.3389/fvets.2020.567258)
Supplement: Supplementary file 1 [file Table_1.pdf]

Supplementary material

Supplementary Table 1. The deaths of 32 cetaceans were due to fishery interactions in the Canary Islands (from January 2000 to December 2018). The table shows the species; diving behavior (D, deep diver or S, shallow diver); stranding date (day.month.year); stranding event (D, dead or A, alive); island (FV, Fuerteventura; GC, Gran Canaria; LNZ, Lanzarote; LP, La Palma; TNF, Tenerife); sex (F, female or M, male); growth development category (calf, juvenile, subadult, adult); gonad maturation (I, immature; M, mature; P, pregnant); body condition (very poor, poor, fair, good); decomposition code (1, very fresh; 2, fresh; 3, moderate autolysis; 4, advanced autolysis; 5, very advanced autolysis); and type of fishing interaction [chronic entanglement; fisherman aggression, and bycatch (longline hook ingestion, aggression during handling (aggression), peracute underwater entrapment (PUE), and dolphins released alive that became stranded and died, owing to PUE lesions (released)].

Case	*Species*	*Diving*	*Stranding date*	*Stranding event*	*Island*	*Sex*	*Growth development category*	*Gonad maturation*	*Body condition*	*Decomposition code*	*Type of Fishing Interaction*
1	*Stenella frontalis*	S	28/04/2000	D	TNF	F	Calf	I	Good	2	Bycatch-PUE
2	*Stenella frontalis*	S	22/03/2001	D	GC	M	Adult	M	Good	5	Longline hook ingestion
3	*Stenella frontalis*	S	26/03/2001	D	GC	M	Adult	M	Good	3	Fisherman aggression
4	*Delphinus delphis*	S	21/05/2001	D	TNF	F	Adult	M	Good	2	Fisherman aggression
5	*Delphinus delphis*	S	21/05/2001	D	TNF	M	Calf	I	Good	2	Fisherman aggression
6	*Stenella coeruleoalba*	S	27/12/2001	A	LNZ	M	Calf	I	Fair	1	Bycatch- returned
7	*Tursiops truncatus*	S	11/05/2002	D	TNF	M	Juvenile	I	Good	2	Bycatch-PUE
8	*Tursiops truncatus*	S	30/05/2002	D	TNF	F	Subadult	I	Good	2	Chronic entanglement

9	*Stenella frontalis*	S	07/05/2004	D	GC	F	Juvenile	I	ND	4	Bycatch-aggression
10	*Mesoplodon europaeus*	D	21/06/2004	D	FV	F	Calf	I	Poor	2	Chronic entanglement
11	*Stenella frontalis*	S	11/04/2005	D	TNF	M	Adult	M	Good	2	Bycatch-aggression
12	*Stenella coeruleoalba*	S	16/08/2005	A	GC	F	Juvenile	I	Fair	2	Bycatch-returned
13	*Stenella frontalis*	S	12/03/2007	D	TNF	F	Adult	M	Good	2	Fisherman aggression
14	*Globicephala macrorhynchus*	D	26/12/2007	A	GC	F	Calf	I	Fair	1	Chronic entanglement
15	*Stenella frontalis*	S	23/03/2008	D	TNF	F	Juvenile	I	Good	3	Fisherman aggression
16	*Balaenoptera acutorostrata*	S	09/04/2009	D	GC	F	Calf	I	Very poor	2	Chronic entanglement
17	*Stenella coeruleoalba*	S	09/03/2012	D	LNZ	F	Adult	M	Good	2	Bycatch-PUE

18	*Stenella frontalis*	S	05/04/2012	D	FV	M	Adult	M	Fair	2	Longline hook ingestion
19	*Balaenoptera acutorostrata*	S	01/04/2013	D	FV	M	Calf	I	Fair	2	Chronic entanglement
20	*Stenella frontalis*	S	02/09/2013	D	FV	M	Adult	M	Fair	5	Longline hook ingestion
21	*Stenella frontalis*	S	19/02/2014	D	LP	F	Adult	M	Fair	2	Bycatch-aggression
22	*Stenella frontalis*	S	20/03/2015	D	TNF	M	Adult	M	Fair	3	Bycatch-aggression
23	*Stenella frontalis*	S	19/12/2015	A	GC	M	Adult	M	Fair	3	Bycatch-returned
24	*Globicephala macrorhynchus*	D	29/12/2015	D	GC	M	Calf	I	ND	5	Chronic entanglement
25	*Stenella coeruleoalba*	S	22/03/2016	D	FV	M	Juvenile	I	Poor	3	Bycatch-PUE
26	*Delphinus delphis*	S	04/06/2016	D	GC	F	Juvenile	M	Fair	2	Bycatch-aggression

27	*Stenella frontalis*	S	02/03/2017	D	TNF	F	Subadult	M	Fair	3	Bycatch-aggression
28	*Stenella coeruleoalba*	S	12/03/2017	D	TNF	M	Subadult	M	Poor	3	Bycatch-PUE
29	*Stenella frontalis*	S	21/03/2017	D	TNF	M	Adult	M	Fair	3	Longline hook ingestion
30	*Stenella coeruleoalba*	S	27/03/2017	D	FV	M	Subadult	I	Fair	3	Bycatch-PUE
31	*Stenella frontalis*	S	04/04/2017	D	TNF	M	Adult	M	Fair	3	Longline hook ingestion
32	*Delphinus delphis*	S	06/09/2018	D	FV	M	Adult	M	Fair	2	Bycatch-aggression
