# Supplementary material for: Retrospective Study of Fishery Interactions in Stranded Cetaceans, Canary Islands
Source: Front Vet Sci. 2020 Oct 21;7:567258. doi: 10.3389/fvets.2020.567258 (PMC7641611; doi:10.3389/fvets.2020.567258)
Supplement: Supplementary file 2 [file Table_2.pdf]

Supplementary Table 2. Gross findings in stranded cetaceans that died because of fishery interactions (chronic entanglement, aggression, or bycatch) (n = 32). For each case, the fishing category, decomposition code, type of stranding event, and lesions found in various organs are shown. The presence of lesions is indicated by an "X" and the absence of lesions is indicated by the symbol "-." NE: not evaluated; ND: Not determined.

													Bycatch (n=21)																						
		**Entanglement (n=6)**						**Aggression (n=5)**					**Hook ingestion (n=5)**					**PUE (n=6)**						**Aggression during handling (n=7)**							**Returned (n=3)**		
	Cases	**8**	**10**	**14**	**16**	**19**	**24**	**3**	**4**	**5**	**13**	**15**	**2**	**18**	**20**	**29**	**31**	**1**	**7**	**17**	**25**	**28**	**30**	**9**	**11**	**21**	**22**	**26**	**27**	**32**	**6**	**12**	**23**
	Decomposition code	2	2	1	2	2	5	3	2	2	2	3	5	2	5	3	3	2	2	2	3	3	3	4	2	2	3	2	3	2	1	2	3
Stranding event	Stranding alive	-	-	X	-	-	-	-	-	-	-	-	-	-	-	-	-	-	-	-	-	-	-	-	-	-	-	-	-	-	X	X	X
	Stranding death	X	X	-	X	X	X	X	X	X	X	X	X	X	X	X	X	X	X	X	X	X	X	X	X	X	X	X	X	X	-	-	-
	Fishing gears attached	-	X	X	X	-	X	-	-	-	-	-	X	X	X	X	X	-	-	-	-	-	-	-	-	-	-	-	-	-	-	X	X
Body condition	Poor-very poor	-	X	-	X	-	-	-	-	-	-	-	-	-	-	-	-	-	-	-	X	X	-	-	-	-	-	-	-	-	-	-	-
	Good-fair	X	-	X	-	X	-	X	X	X	X	X	X	X	X	X	X	X	X	X	-	-	X	-	X	X	X	X	X	X	X	X	X
	NE	-	-	-	-	-	X	-	-	-	-	-	-	-	-	-	-	-	-	-	-	-	-	X	-	-	-	-	-	-	-	-	-
Skin	Net impressions over the body	X	X	X	X	X	X	-	-	-	-	-	-	X	-	X	-	X	X	X	X	-	-	-	X	-	X	-	-	X	-	-	X
	Net cuts in pectoral flippers	-	-	-	-	-	-	-	-	-	-	-	-	-	-	X	X	X	-	-	X	X	X	-	-	X	-	X	-	-	-	-	X
	Net cuts in head	-	-	-	X	-	-	-	-	-	-	-	-	X	-	X	X	X	X	X	-	X	X	X	X	X	X	X	X	X	-	X	X

	Net cuts over the body	-	X	-	-	-	-	-	-	-	-	-	-	X	-	X	X	-	-	X	X	-	X	-	X	X	X	X	-	X	X	X	X
Subcutaneous	Hematoma	-	-	-	-	X	X	X	X	X	X	X	-	X	-	X	X	-	-	-	-	-	-	X	X	X	X	X	X	-	-	-	-
Skeletal muscle	Hemorrhages	-	-	-	X	X	-	X	X	X	X	X	-	X	-	-	X	-	-	-	-	X	-	-	X	X	X	X	X	X	-	-	-
Bones	Mandibles fracture	-	-	-	-	-	-	-	-	-	-	-	X	-	-	-	X	-	-	-	X	-	X	-	-	-	-	X	-	X	X	X	-
	Maxilla fracture	-	-	-	-	-	-	-	-	-	-	-	X	-	-	-	-	-	-	-	X	-	X	-	-	-	-	X	-	-	X	X	-
	Neurocranium fracture	-	-	-	-	-	-	-	-	-	-	-	-	-	-	-	-	-	-	-	-	-	-	-	-	-	-	-	X	X	-	-	-
	Tympanic fracture	-	-	-	-	-	-	-	-	-	-	-	-	-	-	-	-	-	-	-	-	-	-	-	-	-	-	-	-	-	X	-	-
	Vertebrae fracture	-	-	-	-	-	-	-	X	X	-	-	-	-	-	-	-	-	-	-	-	-	-	-	-	-	-	X	-	-	-	-	-
	Rib fracture	-	-	-	-	-	-	-	-	-	-	-	-	-	-	-	-	-	-	-	-	-	-	-	-	-	-	-	-	-	-	X	-
	Teeth lost/fractured	-	-	-	-	-	-	X	X	-	-	-	-	-	-	X	-	-	X	-	X	-	X	-	-	X	-	X	-	X	X	X	-
Digestive tract	Esophagus Fresh/undigested prey	-	-	-	-	-	X	X	-	-	X	X	-	-	-	-	-	X	X	X	-	-	-	-	-	-	X	-	X	-	-	-	-
	Stomach Fresh/undigested prey	-	X	-	-	-	-	-	X	-	X	X	X	-	-	X	-	X	X	X	-	-	-	X	X	-	X	X	X	-	-	-	-

	Digested content	-	-	-	-	-	-	-	-	-	-	-	-	-	-	-	X	-	-	-	-	X	-	-	-	-	-	-	-	-	X	X	X
	Empty/ND	X	-	X	X	X	-	-	-	X	-	-	-	X	X	-	-	-	-	-	X	-	X	-	-	-	-	-	-	X	-	-	-
Bubbles	Lymphatic vessels	-	-	-	-	-	-	-	-	-	X	-	-	-	-	-	-	X	-	-	-	-	-	-	X	-	-	-	-	X	-	-	-
	Blood vessels	-	X	-	-	-	-	-	-	-	-	-	-	-	-	-	X	X	X	X	X	-	X	-	X	-	-	X	X	X	-	-	X
Blood in cavities	Hemothorax	-	-	-	-	X	-	-	X	X	-	X	-	-	-	X	X	-	-	-	-	-	-	-	-	-	X	-	-	-	-	-	-
	Hemoabdomen	-	-	-	-	-	-	-	-	-	-	-	-	-	X	X	X	-	-	-	X	-	-	-	-	-	X	-	-	-	-	-	-
	Hemopericardium	-	-	-	-	-	-	-	-	-	-	-	-	-	-	-	-	-	-	-	-	-	-	-	-	-	X	-	-	-	-	-	-
Lungs	Hyperinflated	-	-	-	X	-	-	-	-	-	-	-	-	-	X	-	X	-	-	X	X	-	-	-	-	X	X	X	X	X	X	X	X
	Hemorrhagic parenchyma	-	-	-	-	-	-	-	X	X	-	X	-	-	-	-	-	X	-	-	-	-	-	-	-	-	X	X	-	-	X	-	-
	Subpleural hemorrhage	-	-	-	-	-	-	X	X	X	-	X	-	-	-	-	-	X	X	X	-	-	-	-	-	-	-	-	-	-	-	-	-
	Tracheal or bronchial edema	-	-	-	X	X	-	-	-	-	-	-	-	-	-	-	-	-	-	-	-	-	-	-	-	-	-	X	-	X	-	-	-
	Rib impressions	-	-	-	-	-	-	-	-	-	-	-	-	-	-	-	-	-	-	-	-	-	-	-	-	-	-	-	-	-	-	X	X
	Rupture of the parenchyma	-	-	-	-	-	-	-	X	X	-	X	-	-	-	-	-	-	-	-	-	-	-	-	X	-	-	-	-	-	-	-	-

Heart	Hemopericardium	-	-	-	-	-	-	-	-	-	-	-	-	-	-	X	-	-	-	-	-	-	-	-	-	-	-	-	-	-	-	-	-
	Hemorrhages	-	-	-	-	-	-	-	-	-	-	-	-	-	-	-	-	-	-	-	-	-	-	-	-	-	X	-	-	-	-	-	-
	Vascular changes in valves	-	-	X	X	-	-	-	-	-	-	-	-	-	-	-	-	-	-	-	-	-	-	-	-	-	-	-	-	-	-	-	-
Large vessels	Aorta vascular changes	-	-	-	X	-	-	-	-	-	-	-	-	-	-	X	-	-	-	-	-	-	X	X	X	-	X	X	-	-	-	-	-
	Rete mirabile vascular changes	-	-	-	-	-	-	-	-	-	-	-	-	-	-	-	-	-	-	-	-	-	-	-	-	-	X	X	-	-	-	-	-
Kidney	Hemorrhages	-	-	-	-	-	-	-	-	-	-	-	-	-	-	-	-	-	-	-	-	-	-	-	-	-	-	X	-	-	-	-	-
	Retroperitoneal emphysema	-	X	-	-	-	-	-	-	-	-	-	-	-	-	-	X	-	-	X	X	-	-	-	X	-	X	-	-	-	-	-	-
Lymphatic	Lymph in vessels	-	-	-	-	-	-	-	X	-	X	-	-	-	-	-	-	X	X	X	-	-	-	-	X	-	X	X	X	-	-	-	-
Brain	Meningeal hemorrhages	-	-	-	-	-	-	-	-	-	X	-	-	-	-	-	-	-	-	-	-	-	-	-	-	-	-	X	X	X	-	-	-
	Parenchymal hemorrhages	-	-	-	-	-	-	-	-	-	-	-	-	-	-	-	-	-	-	-	-	-	-	-	-	-	-	-	X	-	-	-	-
