# Supplementary material for: Retrospective Study of Fishery Interactions in Stranded Cetaceans, Canary Islands
Source: Front Vet Sci. 2020 Oct 21;7:567258. doi: 10.3389/fvets.2020.567258 (PMC7641611; doi:10.3389/fvets.2020.567258)
Supplement: Supplementary file 3 [file Table_3.pdf]

Supplementary Table 3. Histological findings in stranded cetaceans, which died because of fishery interactions, and evaluated with decomposition codes 1–3 (n = 28). For each case, the severity of pathological findings in skeletal and cardiac muscle (L, mild; M, moderate; S, severe); as well as the presence of lesions in the lung, liver, kidney, adrenal gland, blood vessels, and brain (X, presence; -, absence); and gonad maturation (M, mature; I, immature), were detailed and compared upon the availability of samples. NE = appear in not evaluated organs of the studied cases.

												Bycatch (n=18)														
		**Entanglement (n=5)**					**Aggression (n=5)**					**Hook ingestion**			**PUE**						**Aggression during handling**						**Returned**		
	Cases	**8**	**10**	**14**	**16**	**19**	**3**	**4**	**5**	**13**	**15**	**18**	**29**	**31**	**1**	**7**	**17**	**25**	**28**	**30**	**11**	**21**	**22**	**26**	**27**	**32**	**6**	**12**	**23**
	Decomposition code	2	2	1	2	2	3	2	2	2	3	2	3	3	2	2	2	3	3	3	2	2	3	2	3	2	1	2	3
**Skeletal muscle**	Acute degenerative changes	-	L	L	M	L	-	L	NE	M	M	M	L	-	S	NE	L	L	L	M	L	M	L	S	M	M	L	L	L
	Atrophy	-	M	-	M	L	-	L	NE	-	-	-	-	-	-	NE	L	-	-	-	-	-	-	L	L	-	-	-	-
	Hemorrhages	-	-	-	-	-	-	-	NE	-	X	X	-	-	-	NE	-	-	-	-	X	X	-	X	X	-	-	-	-
**Lungs**	Alveolar edema	X	X	-	-	-	X	X	-	X	X	X	X	X	-	X	X	X	-	X	-	X	-	X	X	X	-	NE	-
	Emphysema	X	-	-	X	X	-	X	X	-	X	-	X	X	X	X	-	X	X	X	X	-	-	X	X	X	X	NE	X
	Muscular bronchiolar sphincter contraction	-	-	-	-	-	X	-	X	X	X	X	X	-	-	X	-	-	-	X	X	-	-	-	X	-	-	NE	-
	Hemorrhages	-	X	-	-	-	X	X	X	X	-	-	X	X	X	X	X	-	X	-	X	-	X	X	X	-	X	NE	X
**Heart**	Acute degenerative changes	L	L	L	L	L	-	NE	-	L	-	L	M	M	NE	L	L	-	L	L	-	-	-	L	-	M	NE	NE	-
	Hemorrhages	-	-	-	X	X	-	NE	-	-	-	-	-	-	NE	-	-	-	-	-	X	-	-	-	X	X	NE	NE	-

**Liver**	Intracytoplasmic hyaline globules	X	-	X	X	X	NE	NE	X	X	-	-	X	X	X	X	X	NE	X	NE	-	X	NE	X	X	X	-	-	X
**Adrenal gland**	Hemorrhages	X	X	-	X	X	NE	-	X	X	-	-	-	X	X	-	X	NE	-	-	-	-	-	-	-	X	-	X	-
**Blood vessels**	Leukocytosis	X	X	-	X	X	X	X	-	X	-	X	X	X	X	X	X	-	X	X	-	X	X	X	X	X	X	-	X
	Intravascular coagulation	-	X	X	-	-	-	-	-	-	-	-	-	X	-	-	-	-	X	-	-	X	X	X	X	X	-	-	-
	Intravascular clear spaces	X	X	X	X	X	X	X	X	X	X	X	X	X	X	X	X	X	X	X	X	X	X	X	-	X	X	X	X
**Reproductive system**	Gonadal maturity	M	M	I	I	I	M	M	I	M	I	M	M	M	I	I	M	I	M	I	M	M	M	M	M	I	I	I	M
**Kidney**	Membranous glomerulonephritis	-	-	-	-	-	X	X	-	-	-	X	-	-	-	-	X	-	-	-	-	-	-	-	X	-	-	-	-
	Hyaline cast	-	X	-	-	-	-	-	-	-	-	X	-	X	-	-	-	-	X	-	-	X	-	-	-	X	-	-	-
	Pigmentary tubulonephrosis	-	-	-	-	-	-	-	-	X	-	-	-	-	-	X	X	-	-	-	-	-	-	-	X	-	X	-	-
	Hemorrhages	X	-	-	-	X	-	-	-	-	-	-	-	-	-	-	-	-	-	-	-	-	-	-	X	-	-	-	-
**Brain**	Perivascular edema	X	-	NE	-	-	X	X	X	-	NE	-	-	X	-	-	X	-	-	-	-	-	-	X	-	X	X	-	-
	Hemorrhages	X	-	NE	X	-	-	-	-	X	NE	-	X	X	X	X	-	-	-	-	X	-	-	X	-	X	X	-	-

	Meningitis/encephalitis	X	-	NE	-	-	X	-	-	-	NE	-	-	-	-	-	-	-	-	-	-	-	-	-	-	-	-	X	-
	Perivascular cuffs	-	-	NE	-	-	X	X	-	-	NE	-	-	-	-	-	-	-	-	X	-	X	-	-	X	X	-	-	-
	Glial nodules	X	-	NE	X	X	-	-	-	-	NE	-	X	-	-	-	-	-	-	X	-	X	-	-	X	-	-	-	-
